# Artificial intelligence–assisted ultrasound imaging in hemophilia: research, development, and evaluation of hemarthrosis and synovitis detection

**DOI:** 10.1016/j.rpth.2024.102439

**Published:** 2024-05-09

**Authors:** Azusa Nagao, Yusuke Inagaki, Keiji Nogami, Naoya Yamasaki, Fuminori Iwasaki, Yang Liu, Yoichi Murakami, Takahiro Ito, Hideyuki Takedani

**Affiliations:** 1 Department of Blood Coagulation, Ogikubo Hospital, Tokyo, Japan; 2 Department of Rehabilitation Medicine, Nara Medical University, Nara, Japan; 3 Department of Pediatrics, Nara Medical University, Nara, Japan; 4 Department of Transfusion Medicine, Hiroshima University, Hiroshima, Japan; 5 Division of Hematology and Oncology, Kanagawa Children’s Medical Center, Kanagawa, Japan; 6 Clinical Development Division, Chugai Pharmaceutical Co, Ltd, Tokyo, Japan; 7 Medical Affairs Division, Chugai Pharmaceutical Co, Ltd, Tokyo, Japan; 8 Department of Rehabilitation, National Hospital Organization Tsuruga Medical Center, Fukui, Japan

**Keywords:** AI (artificial intelligence), hemophilia, hemarthrosis, synovitis, ultrasound imaging

## Abstract

**Background:**

Joint bleeding can lead to synovitis and arthropathy in people with hemophilia, reducing quality of life. Although early diagnosis is associated with improved therapeutic outcomes, diagnostic ultrasonography requires specialist experience. Artificial intelligence (AI) algorithms may support ultrasonography diagnoses.

**Objectives:**

This study will research, develop, and evaluate the diagnostic precision of an AI algorithm for detecting the presence or absence of hemarthrosis and synovitis in people with hemophilia.

**Methods:**

Elbow, knee, and ankle ultrasound images were obtained from people with hemophilia from January 2010 to March 2022. The images were used to train and test the AI models to estimate the presence/absence of hemarthrosis and synovitis. The primary endpoint was the area under the curve for the diagnostic precision to diagnose hemarthrosis and synovitis. Other endpoints were the rate of accuracy, precision, sensitivity, and specificity.

**Results:**

Out of 5649 images collected, 3435 were used for analysis. The area under the curve for hemarthrosis detection for the elbow, knee, and ankle joints was ≥0.87 and for synovitis, it was ≥0.90. The accuracy and precision for hemarthrosis detection were ≥0.74 and ≥0.67, respectively, and those for synovitis were ≥0.83 and ≥0.74, respectively. Analysis across people with hemophilia aged 10 to 60 years showed consistent results.

**Conclusion:**

AI models have the potential to aid diagnosis and enable earlier therapeutic interventions, helping people with hemophilia achieve healthy and active lives. Although AI models show potential in diagnosis, evidence is unclear on required control for abnormal findings. Long-term observation is crucial for assessing impact on joint health.

## Essentials

• Early diagnosis of joint bleeding in people with hemophilia is essential for improved outcomes.
• Artificial intelligence may contribute to appropriate use and uptake of ultrasound imaging.
• The artificial intelligence algorithm excelled in detecting hemarthrosis and synovitis.
• Results were consistent between people 10 and 60 years of age and across both hemophilia A and B.

## Graphical abstract

**Figure:**
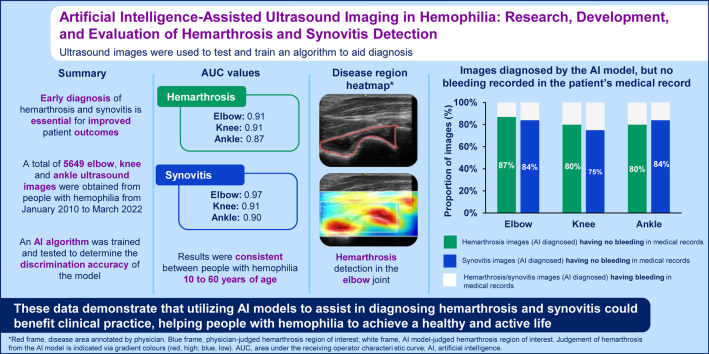

## 1 Introduction

Congenital hemophilia is a rare X-linked bleeding disorder characterized by a deficiency of clotting factor (F)VIII (hemophilia A [HA]) or FIX (hemophilia B [HB]). People with congenital hemophilia are prone to recurrent bleeding, most commonly into joints, known as hemarthrosis [1]. Elbows, knees, and ankles account for approximately 80% of joint bleeds in people with hemophilia [1]. Over time, such bleeding leads to synovial hypertrophy and angiogenesis, followed by cartilage damage, and eventually hemophilic arthropathy and joint destruction [1,2].

The decision on how to treat a bleed is usually made by subjective evaluation by the patient, based on pain, feeling of heat/aura, or swelling. However, owing to the potential absence of the symptoms that accompany more severe, harmful bleeds, smaller bleeds may not be identified and treated, leading to joint damage that only becomes apparent during clinical evaluation [3].

Magnetic resonance imaging (MRI) data have shown joint damage in a proportion of people with hemophilia who had no evidence of joint bleeds, with the damage speculated to be related to chronic subclinical bleeding [4]. Early diagnosis of joint bleeding and synovitis, including asymptomatic occurrences and appropriate therapeutic interventions, are expected to help with the achievement of a healthy and active life for people with hemophilia.

Currently, MRI, standard radiography (X-ray), and ultrasound imaging are used for evaluating joints, but there are difficulties associated with utilizing ultrasound imaging and MRI in day-to-day clinical practice, as well as difficulties in using X-ray for synovitis detection. Although X-ray is a widely available imaging tool used at most medical institutions, it is not able to identify signs of early-stage joint disease in people with hemophilia [5]. Thus, the radiological Pettersson score cannot be considered a reliable tool to detect subclinical joint impairment [6]. Additionally, although MRI is considered the gold standard and provides detailed information that is useful for diagnosing abnormal joints, it cannot routinely be used in clinical practice due to high costs and a potential need for sedation in children [2,7]. A good correlation has been shown between the ability of MRI and ultrasound imaging to detect joint bleeds [8,9], and ultrasound has the advantages of being quicker, costing less, being suitable for use in point-of-care settings, and not requiring sedation for children [2,[10], [11], [12], [13], [14], [15]]. However, accurate diagnosis of hemarthrosis and synovitis depends on the skills and experience of the examiner [[11], [12], [13], [14], [15]].

If hemarthrosis and synovitis could be easily diagnosed in an outpatient setting, treatment decisions could be guided to ensure that bleeds, even those that are asymptomatic, are treated effectively. This would in turn limit joint damage, resulting in improved quality of life over the long term [16].

The introduction of artificial intelligence (AI) models for diagnosing medical conditions could reduce the burden on healthcare professionals and prevent oversights by alleviating the problem that traditional diagnoses are examiner-dependent [17]. There has already been success in utilizing AI for endoscopy, chest X-rays, and pathological examinations [[18], [19], [20], [21]]. It has been reported that various AI models can facilitate clinical care for professionals, improving diagnosis and treatment for people with hemophilia [22]. Examples of how AI has been used to aid diagnostic imaging are illustrated by the mammary gland, thyroid, and cardiac ultrasonography fields [[23], [24], [25], [26]]. Data suggest that sensitivity and specificity of AI diagnostic models are comparable with those of experienced physicians [25]. Using noninvasive ultrasound images and an AI model to provide a diagnosis that is comparable with an expert, regardless of the healthcare professional, would be a remarkable advancement in the medical diagnostic field.

Herein, we research, develop, and evaluate the diagnostic precision of our AI algorithm to estimate the presence or absence of hemarthrosis and synovitis using joint ultrasound images obtained and archived through the diagnosis of hemarthrosis and synovitis in people with hemophilia.

## 2 Methods

### 2.1 Study design and data collection in people with hemophilia

This multicenter, noninterventional, observational study used retrospectively collected ultrasound images from people with congenital HA or HB. Images were taken from the elbow, knee, and ankle joints in people with congenital HA or HB, with or without inhibitors between January 1, 2010, and March 31, 2022, and were collected from 5 different hemophilia treatment centers in Japan.

Established information such as disease name (including presence/absence of inhibitors), month and year of birth, date of imaging, joint site (elbows, knees, and ankles), and bleeding records within 6 months of imaging was collected for each participant.

Participants were included in the study if they had either congenital HA or HB and were undergoing ultrasound imaging between January 1, 2010, and March 31, 2022, and were only included if they did not express their intention to refuse participation, or opt out.

The protocol was approved by institutional review boards or ethics committees at each center. The trial was conducted in accordance with the Declaration of Helsinki, the Act on the Protection of Personal Information, the Ethical Guidelines for Medical and Biological Research involving Human Subjects, and the International Conference on Harmonization Guidelines for Good Clinical Practice.

### 2.2 Ultrasound imaging annotation procedure for hemarthrosis and synovitis

The contract research organization Hitachi, Ltd. assigned anonymous enrollment numbers to the joint ultrasound images that were provided to the study executive committee, which consisted of 1 physician and 2 orthopedists, all of whom are experts in the hemophilia specialty (the physician had 5 years of experience in ultrasound diagnostics and the orthopedists had 10 and 11 years of experience, respectively, in ultrasound diagnostics). Two members of the study executive committee, 1 physician and 1 orthopedic surgeon, independently annotated the joint ultrasound images using previously agreed annotation criteria (the annotation criteria were decided by the 3 members of the committee at an in-person meeting prior to the start of the study). If the judgments of these 2 members of the committee differed from one another, the third member (the orthopedist) assessed the images and selected the most appropriate diagnosis. If a final decision could not be made at that point, the precision of diagnostic imaging was standardized by determining the most appropriate annotation by discussion among all 3 members of the committee. In this study, we decided to determine the discrimination accuracy for images of fully matured joints to evaluate the effectiveness of the AI model. Therefore, we excluded images from the analysis data that were determined by physicians to be growing joints. In addition, images of arthropathy without bleeding were excluded from the analysis data, as it was necessary to construct the AI model based on standard joint shapes to more accurately detect microbleeding.

The joint ultrasound images were subsequently stratified by the presence or absence of hemarthrosis and synovitis before being randomly divided into training and test datasets using a ratio of 7:3, which is the most common ratio used in the deep learning industry [27,28]. As such, of the joint ultrasound images to be used for analysis of hemarthrosis and synovitis, 70% were assigned as training data and used for training the AI models and the remaining 30% were used for validating and testing the models.

In the training phase, the final annotated images were used to perform neural network learning. In total, 6 models were created; 2 models (1 for hemarthrosis and 1 for synovitis) were created for each joint site (elbow, knee, and ankle). Each of the models was constructed using essentially the same framework.

### 2.3 AI algorithm construction procedure

**Figure 1:**
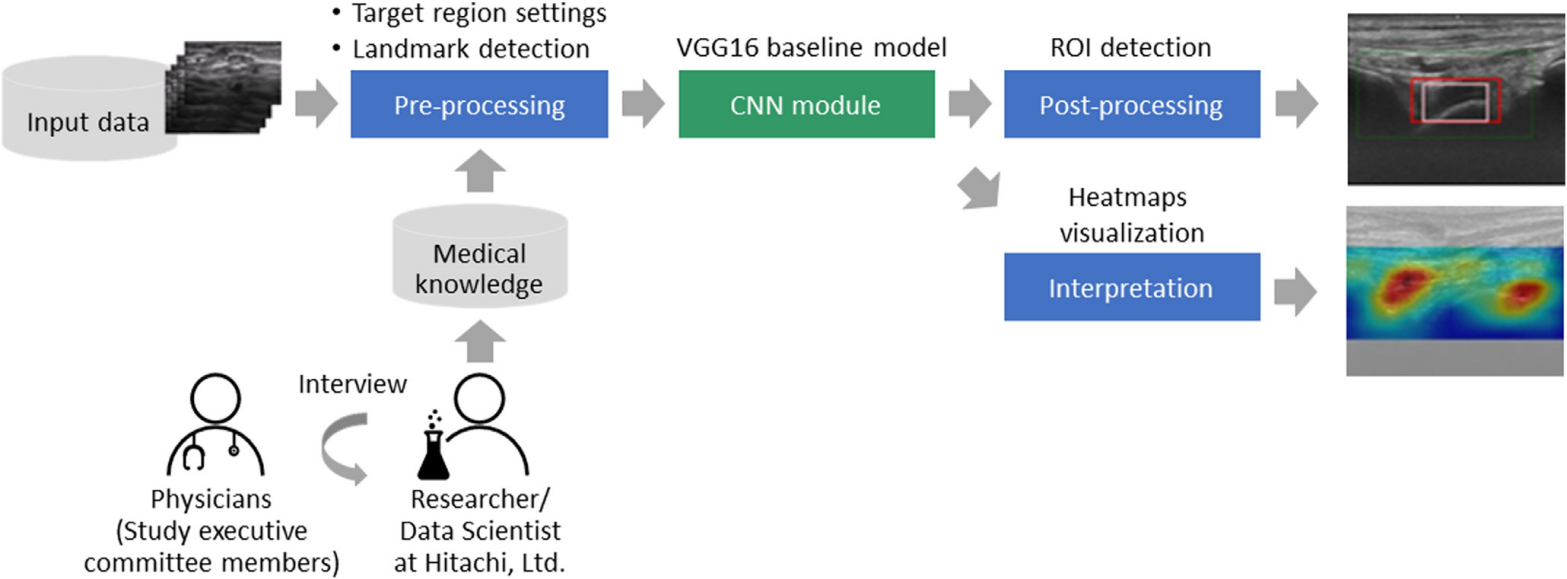
Overall framework of the hemarthrosis and synovitis detection algorithms. CNN, convolutional neural network; ROI, region of interest.

The overall framework of the algorithm is illustrated in Figure 1 and consisted of various steps from preprocessing to model interpretation. The preprocessing module removed noise-like detection information, such as extracting joint regions, and removed text information. As the bone is a robust landmark, detection was performed on the model of deconvolution using the bone surface annotations, previously provided by the study executive committee, for the identification of the analysis area. The convolutional neural network (CNN) module utilized the VGG16 baseline model [29]. This CNN module extracted features to detect the presence/absence of hemarthrosis and synovitis. In the postprocessing module, the pattern of the detection area by the model of deconvolution was used to set regions of interest (ROIs). Only ROIs with a high degree of certainty, which was assessed by the likelihood of the model, remained and were combined with the original image. In the interpretation module, heatmaps of intermediate-level features were generated and visualized by gradient-weighted Class Activation Mapping (Grad-CAM) [30]. In the heatmap output, the lesion site ROI estimated by the AI model was enclosed by a rectangle, and the contribution of the AI model–judged result of hemarthrosis or synovitis was displayed using a gradient, with red illustrating the highest contribution and blue showing the lowest contribution.

In the test phase, the AI models used the inputted joint ultrasound image to extract abnormal areas of interest and identify the presence/absence of hemarthrosis and/or synovitis. Diagnoses made by the study executive committee and the AI algorithm regarding the presence/absence of hemarthrosis and synovitis will be tabulated, and the area under the receiver operating characteristic curve of the outcome of the AI-based diagnostic precision against the researcher-based diagnoses will be evaluated.

### 2.4 Endpoints

The primary endpoint was the area under the receiver operating characteristic curve (AUC) for the diagnostic precision of the AI model to diagnose hemarthrosis and synovitis in elbow, knee, and ankle joints. Additional endpoints examined the accuracy, precision, sensitivity, and specificity of the AI model to diagnose hemarthrosis and synovitis in elbow, knee, and ankle joints by age and hemophilia type (A or B).

Additional analysis was performed to evaluate what proportion of images identified as displaying hemarthrosis or synovitis by the AI model came from people with no documentation of bleeding in their medical records within the 6 months prior to the image being acquired.

### 2.5 Statistical analysis

The sample size was based on the maximum number of joint ultrasound images from the study sites, as the accuracy of AI-based analysis increases with the number of images used to test the models.

The accuracy, sensitivity, specificity, and precision were calculated as follows: true positive (TP), false positive (FP), true negative (TN), and false negative (FN). Accuracy = (TP + TN)/(TP + FP + TN + FN), sensitivity = TP/(TP + FN), specificity = TN/(TN + FP), and precision = TP/(TP + FP). The AUC values were calculated for each of these using the TP and FP rates to assess the performance of the model. An AUC of 1.0 indicates a perfect classification, whereas 0.5 would indicate that the model performs no better than random chance; the higher the AUC value, the better the discriminatory ability of the model. The AUC was calculated using Python version 3.10 (numpy [version 1.23]; scikit-learn [version 1.1]).

## 3 Results

### 3.1 Ultrasound imaging collection in people with hemophilia

At the data cut-off of March 31, 2022, a total of 5649 ultrasound images were collected from the 5 study sites in Japan and assessed by the study executive committee. No participants opted out of the study after enrollment.

Of the ultrasound images collected, 2214 images were excluded from the subsequent analysis as they could not be definitively determined as normal or abnormal, or the bone plane, hemarthrosis, or synovitis could not be annotated according to the predefined criteria. Of the 3435 images annotated and used for this analysis, 2000 showed normal physiology and 1435 had clinical findings. Of these, 1310 showed hemarthrosis and 891 showed synovitis. The images used in this analysis included 464 people with congenital HA and 108 people with congenital HB. Full patient demographics and image characteristics can be found in Table 1.

**Table 1:** Patient demographics and image characteristics used of those used for analysis.

	Elbow	Knee	Ankle
Number of patients	179	202	191
Hemophilia A without inhibitors	144	165	150
Hemophilia A with inhibitors	1	1	3
Hemophilia B without inhibitors	34	36	37
Hemophilia B with inhibitors	0	0	1
Number of joint images	966	1534	935
Annotated joint type			
Normal joints^a^	708	923	369
Hemarthrosis	228	552	530
Synovitis	87	503	301
Age of patient image is taken from (y)			
0-9	0	0	0
10-19	78	115	134
20-29	290	397	324
30-39	227	361	213
40-49	233	386	175
50-59	114	240	81
60-69	20	35	8
70-79	4	0	0
≥80	0	0	0
Disease type of patient image is taken from			
Hemophilia A	763	1225	739
Hemophilia B	203	309	196

a Joints without hemarthrosis/synovitis.

### 3.2 Endpoints

**Figure 2:**
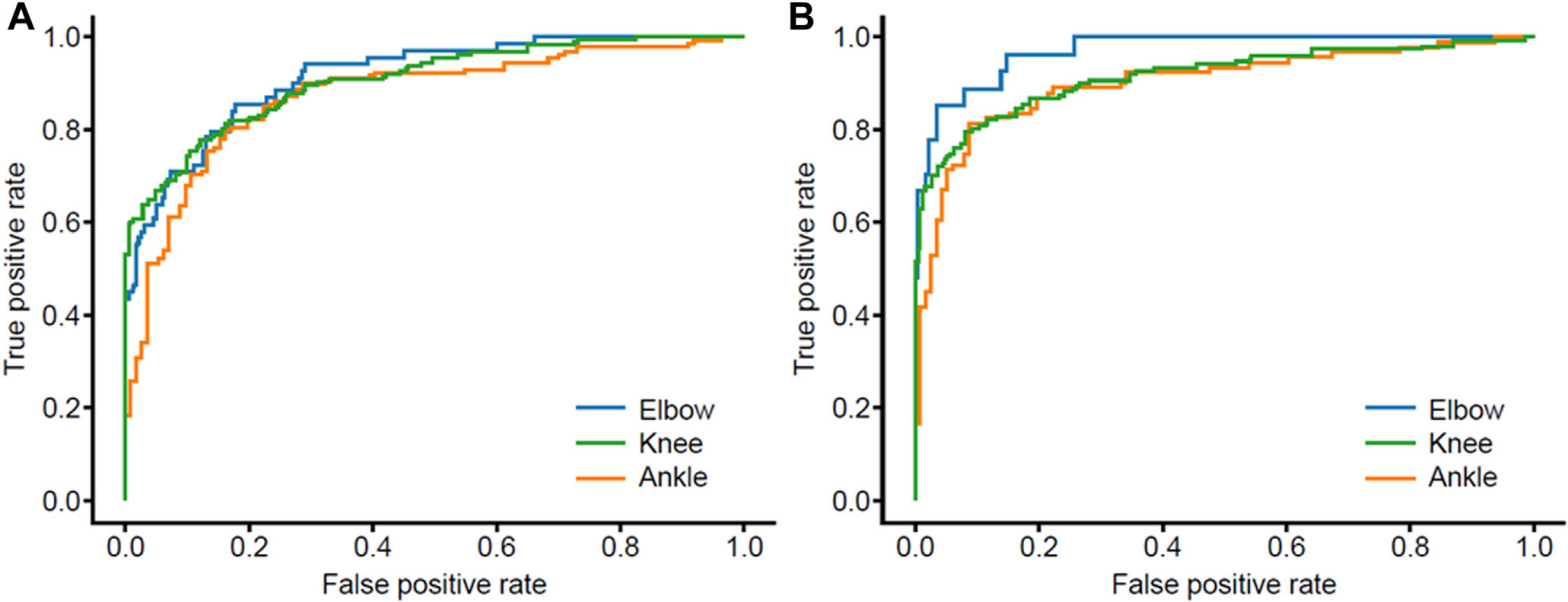
ROC curve for the hemarthrosis and synovitis detection in the elbow, knee, and ankle joints. (A) Hemarthrosis and (B) synovitis detection in the elbow, knee, and ankle joints. ROC, receiver operating characteristic.

The results of the primary endpoint of AUC for the algorithm for hemarthrosis detection in the elbow, knee, and ankle joints were 0.91, 0.91, and 0.87, respectively. The AUC values for the algorithm for the synovitis detection of the elbow, knee, and ankle joints were 0.97, 0.91, and 0.90, respectively (Table 2, Figure 2).

**Table 2:** Mean AUC by age for the AI models of hemarthrosis and synovitis.

	All	10-19 y	20-29 y	30-39 y	40-49 y	50-59 y
Hemarthrosis algorithm, synovitis algorithm						
Elbow	0.91, 0.97 (*N* = 282, 240)	0.94, 0.92 (*N* = 31, 25)	0.91, 0.97 (*N* = 94, 79)	0.90, 1.00 (*N* = 53, 43)	0.93, 0.96 (*N* = 67, 61)	0.93, 0.98 (*N* = 29, 25)
Knee	0.91, 0.91 (*N* = 443, 428)	0.98, 0.94 (*N* = 38, 38)	0.84, 0.95 (*N* = 112, 108)	0.89, 0.91 (*N* = 94, 102)	0.93, 0.91 (*N* = 115, 103)	0.95, 0.91 (*N* = 76, 69)
Ankle	0.87, 0.90 (*N* = 270, 202)	0.85, 0.95 (*N* = 39, 39)	0.82, 0.89 (*N* = 96, 67)	0.91, 0.94 (*N* = 53, 39)	0.90, 0.84 (*N* = 52, 36)	0.91, 0.90 (*N* = 28, 21)

AI, artificial intelligence; AUC, area under the curve.

The results of the secondary endpoints of the accuracy of the algorithm to detect hemarthrosis in the elbow, knee, and ankle joints were 0.87, 0.79, and 0.74, respectively. For precision of the algorithm to detect hemarthrosis in the elbow, knee, and ankle joints, values were 0.77, 0.67, and 0.72, respectively. The values for the accuracy of the algorithm to detect synovitis in the elbow, knee, and ankle joints were 0.95, 0.84 and 0.83, respectively, while for precision, they were 0.80, 0.74, and 0.80, respectively (Table 3).

**Table 3:** Accuracy, precision, sensitivity, and specificity for the hemarthrosis and synovitis algorithms.

	Elbow (*N* = 282, 240)	Knee (*N* = 443, 428)	Ankle (*N* = 270, 202)
Hemarthrosis algorithm, synovitis algorithm			
Accuracy	0.87, 0.95	0.79, 0.84	0.74, 0.83
Precision	0.77, 0.80	0.67, 0.74	0.72, 0.80
Sensitivity	0.68, 0.74	0.86, 0.85	0.92, 0.84
Specificity	0.90, 0.97	0.90, 0.91	0.81, 0.86

The analysis of the hemarthrosis and synovitis algorithms by age group can be seen in Table 2.

The AUC values for the algorithm to detect hemarthrosis in the elbow, knee, and ankle joints in people with HA were 0.92, 0.90, and 0.86 and for people with HB were 0.90, 0.94, and 0.89, respectively. The AUC values for the algorithm to detect synovitis in the elbow, knee, and ankle joints in people with HA were 0.97, 0.90, and 0.88 and for people with HB were 0.98, 0.96, and 0.96, respectively.

**Figure 3:**
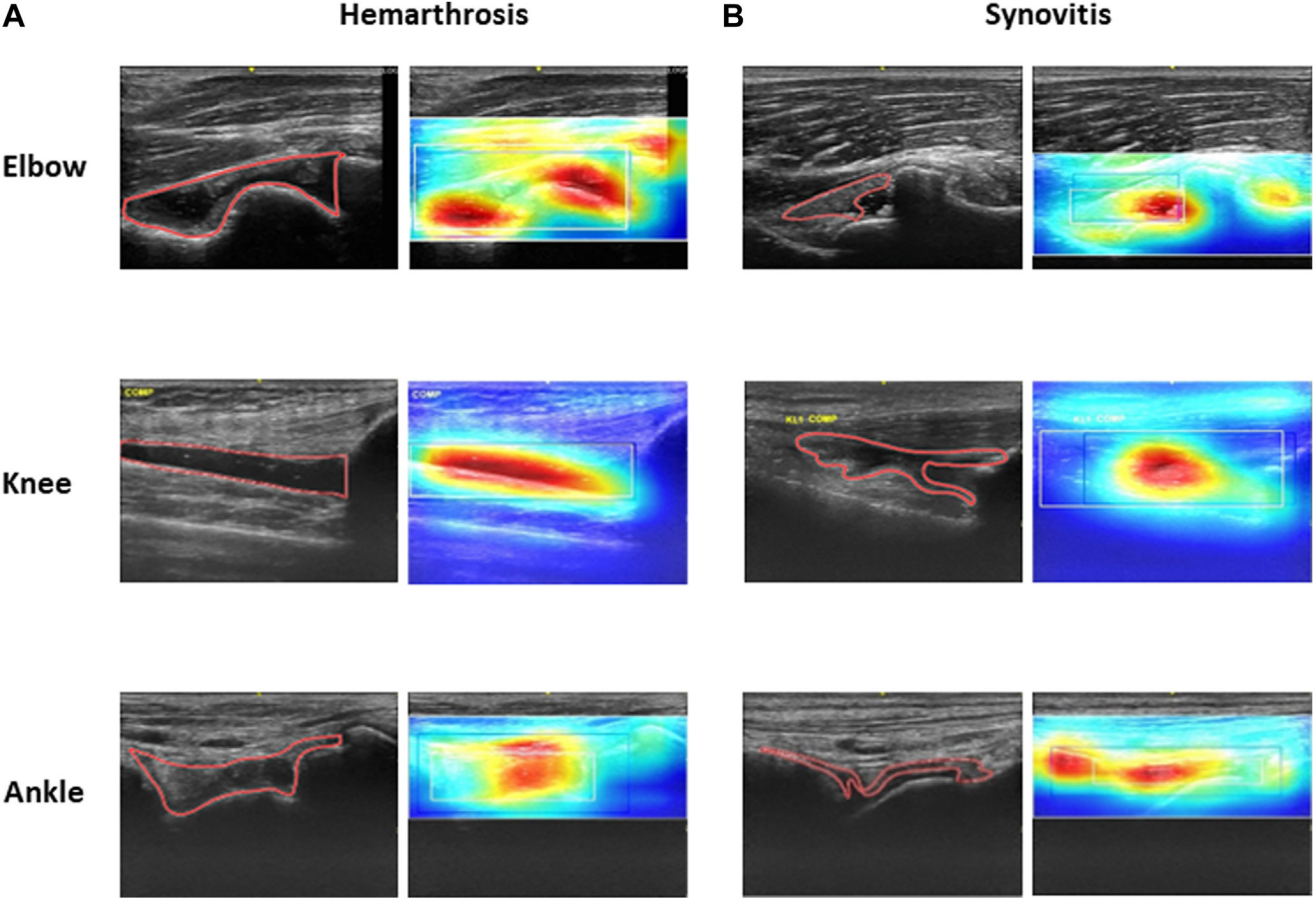
Example successful hemarthrosis and synovitis detection results of the disease region heatmaps in the elbow, knee, and ankle joints. Example (A) hemarthrosis and (B) synovitis detection results of the disease region heatmaps in the elbow, knee, and ankle joints. Comparison of physician's annotation and AI model judged for synovitis areas. The red frame indicates the disease area annotated by the physician. The blue frame indicates physician-judged hemarthrosis or synovitis ROI. The white frame indicates AI model–judged hemarthrosis or synovitis ROI. The judgment of hemarthrosis or synovitis from the AI model is indicated as a gradient color layer (higher factor: red; lower factor: blue). AI, artificial intelligence; ROI, region of interest.

Hemarthrosis and/or synovitis region detection was implemented to show the area of disease. Example ROI detection and heatmaps for hemarthrosis and synovitis are illustrated in Figure 3.

**Figure 4:**
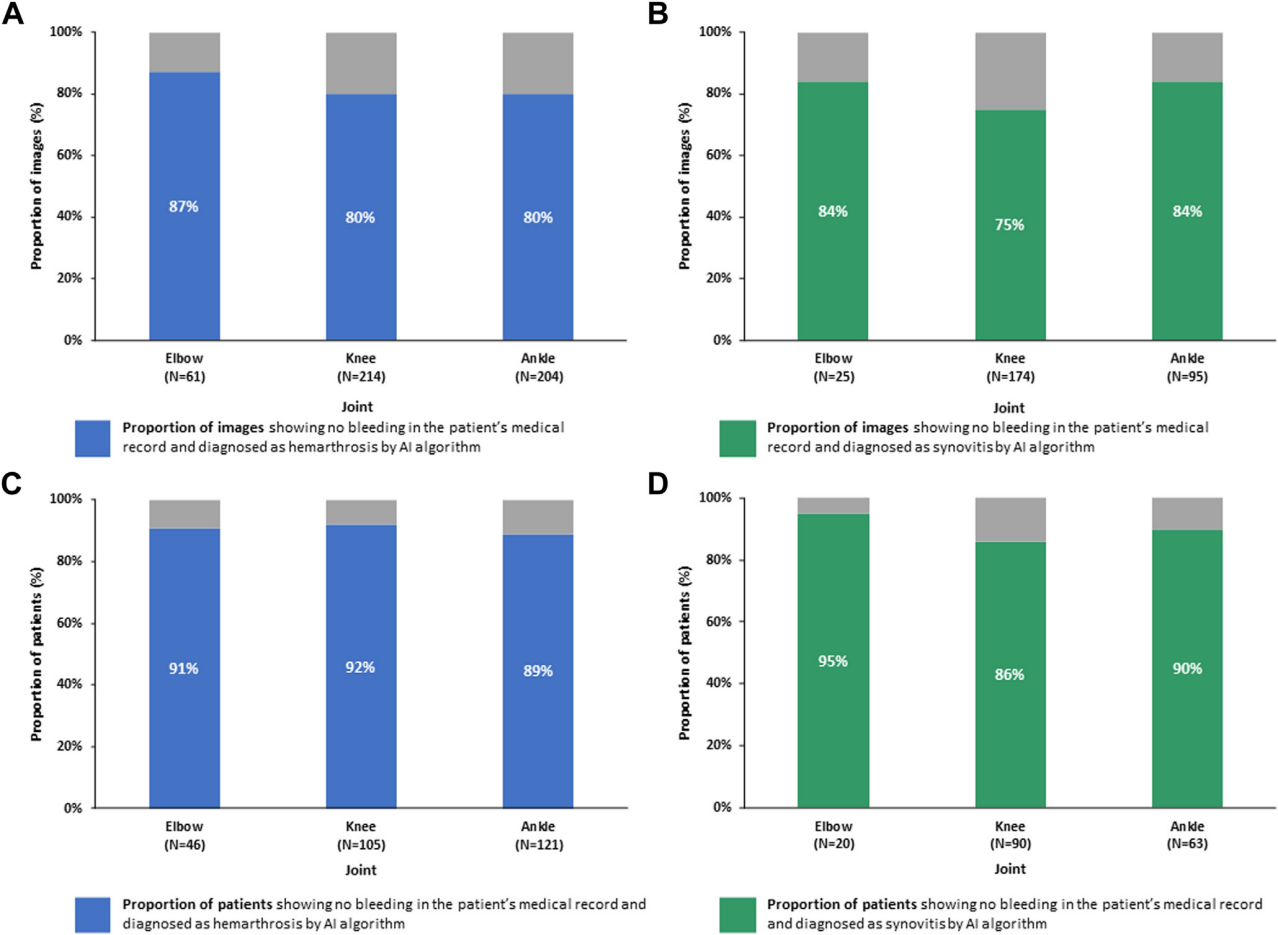
Proportion of images and patients diagnosed as hemarthrosis and synovitis by the AI model with no bleeding recorded in the patient’s medical records. (A) Proportion of images with hemarthrosis identified by the algorithm without bleeding in the patient’s medical records (blue) or with bleeding in the patient’s medical records (gray). (B) Proportion of images with synovitis identified by the algorithm without bleeding in the patient’s medical records (green) or with bleeding in the patient’s medical records (gray). (C) Proportion of patients diagnosed as hemarthrosis and identified by the algorithm without bleeding in the patient’s medical records (blue) or with bleeding in the patient’s medical records (gray). (D) Proportion of patients diagnosed with synovitis and identified by the algorithm without bleeding in the patient’s medical records (green) or with bleeding in the patient’s medical records (gray). AI, artificial intelligence.

The proportion of images that were diagnosed as having hemarthrosis and/or synovitis by the AI model when no bleeding was present in patient’s medical records are outlined in Figure 4. The numbers of joint images from people with no documentation of bleeding in their medical records out of all the images that were judged to show hemarthrosis by the AI algorithm were 53/61 (87%) for the elbow, 171/214 (80%) for the knee, and 164/204 (80%) for the ankle joint. The numbers of joint images from people with documentation of no bleeding in their medical records out of all the images that were judged to show synovitis by the AI algorithm were 21/25 (84%) for the elbow, 131/174 (75%) for the knee, and 80/95 (84%) for the ankle joint.

The number of patients diagnosed with hemarthrosis by the AI algorithm and for whom no bleeding was recorded in their medical records was 42/46 (91%) for the elbow, 97/105 (92%) for the knee, and 108/121 (89%) for the ankle joint (Figure 4). The number of patients diagnosed with synovitis by the AI algorithm and for whom no bleeding was recorded in their medical records was 19/20 (95%) for the elbow, 77/90 (86%) for the knee, and 57/63 (90%) for the ankle joint.

## 4 Discussion

This study researched, developed, and evaluated the diagnostic precision of an AI model to detect the presence/absence of hemarthrosis and synovitis in elbow, knee, and ankle joints. Our study reported an AUC of ≥0.87 for the hemarthrosis algorithm and ≥0.90 for the synovitis algorithm. The ability of the AI model to accurately diagnose hemarthrosis and/or synovitis was consistent between people with HA and HB and relatively consistent between those aged 10 to 60 years.

Whilst prophylactic treatment is widely used in people with hemophilia, joint bleeds still occur. Prevention of bleeding and joint damage to improve health and quality of life is a priority for the care of people with hemophilia [16]. The time taken for the AI model to discriminate and provide a diagnosis is less than 1 second per image, which highlights the potential of using an AI model to provide rapid, accurate diagnoses through ultrasound imaging in an outpatient setting. This would directly contribute to clinical care, as early identification of joint bleeding and synovitis is necessary to optimize treatment strategies and prevent the development of irreversible joint damage.

The feasibility of such an approach has been demonstrated in other fields, including oncology, respiratory medicine, and ophthalmology, with similar AUC values to those produced in this study [[24], [25], [26],^31^]. In a study discriminating between benign and malignant breast mass images, deep learning with CNN had an equal or better performance compared with radiologists (AUC = 0.913 and 0.728–0.845, *P* = .01–.14), respectively [24]. A recent meta-analysis evaluated the diagnostic accuracy of AI algorithms to identify pathology in medical imaging and found that AUC values ranged from 0.864 and 0.937 in respiratory imaging and 0.868 and 0.909 for breast imaging [31]. These data suggest that our AI algorithm may perform as well as AI models in other fields of medicine.

Although direct comparisons cannot be made due to differences in the studies, in hemophilia specifically, Gualtierotti et al. [32] evaluated the diagnostic accuracy of using a computer-aided diagnosis system for the automatic detection of joint effusion in musculoskeletal ultrasound images of the suprapatellar bursa longitudinal scan of the knee; joint recess distensions were detected with an accuracy of 88% (80% sensitivity; 93% specificity). Like the current study, the learning algorithm was based on an object detection framework that was trained to detect the normal and the distended joint recesses [32]. In the present study, all 6 models displayed high accuracy, ranging from 74% for the ankle hemarthrosis algorithm to 95% for the elbow synovitis algorithm, which together with the sensitivities and specificities achieved, demonstrates the potential for this diagnostic approach.

To account for the differences in the accuracy and precision between the different joints evaluated, we conclude that for the ankle joint, there were multiple images in which it was difficult to detect the bleeding site, this resulted in lower accuracy compared with the elbow and knee joints. In addition, the ultrasound images of the knee joint had a thick muscle and fat layer and included more images with unclear bleeding sites than those of the elbow and ankle; we consider that this effect resulted in a decrease in precision compared with that in the elbow and ankle.

To gauge if the AI algorithm provided additional insights into clinical practice, we compared patient bleeding records with the AI algorithm–judged images. Of the 61 elbow joint images for which hemarthrosis was diagnosed by the AI algorithm, there were as many as 53 (87%) images for which no record of bleeding could be confirmed in the patient’s medical records. In addition, of the 46 patients with images of their elbow joint and no bleeding recorded in their medical records, 42 (91%) were diagnosed with hemarthrosis by the AI algorithm. These data suggest that the AI algorithm may have identified bleeding that was originally missed in the patient’s subjective assessment before joint imaging was performed. Similar results were demonstrated for diagnosis of synovitis in all 3 joint types and for hemarthrosis in the knee (80% of images; 92% of patients) and ankle (80% of images; 89% of patients) joints; the minor differences between the percentages of images for the 3 joints could be explained by the elbow joint being nonweight bearing and associated with less pain, and thus, it is possible that fewer patients noticed the bleeding. If the AI algorithm can support the diagnosis of joint images, the patient’s subjective assessment of previously overlooked bleeding and synovitis may be revealed. Accurate, early diagnoses of hemarthrosis and synovitis could reduce the burden on physicians, allow for early therapeutic intervention, and improve patient-specific outcomes.

### 4.1 Study limitations

It is worth noting that although the results from this analysis indicate that AI models may have a role in diagnosing hemarthrosis and synovitis through ultrasound imaging, there is no clear evidence on how much control is needed for the abnormal findings detected and how to intervene. Therefore, future long-term observation is necessary to determine the impact on future joint health. In addition, it has been reported that AI models may have some limitations, which raise operational and ethical issues. As such, AI models should be integrated prudently and reasonably within the practitioner’s workflow [22].

The main reason AUC was chosen as the primary endpoint of this study is that it provides a quantitative assessment of the accuracy of the model, independent of the threshold value. However, there are some limitations; for example, the spatial distribution of the model errors. In the future, the uncertainty evaluation will be considered [33]. Also, whether AUC is the most relevant endpoint for this study will be verified in future prospective clinical studies, including study design.

Although the AI model had a high AUC for the analysis of hemarthrosis and/or synovitis in each joint between 10 and 60 years of age, no images from people aged <10 years and few images from those aged ≥60 years were included in this AI model, which was due to the exclusion of images of growing joints or images of arthropathy without bleeding from this analysis. Ensuring that the AI models are also accurate in these age groups is considered important in the uptake of the model into clinical practice. Relatively low AUC values were observed in some joints in the ≥60 years old group of the hemarthrosis and synovitis models. It is thought that this may be because of a small sample size. Future research should prioritize a bigger sample size, especially in the elderly and pediatric patient populations. Owing to the existence of the epiphyseal line and the large cartilage area, and as the joints develop greatly in those aged <10 years old, it is difficult to identify the articular surface in images from this patient age group. In this study, we decided to analyze images of fully mature joints for the development of the annotation criteria in order to construct an effective AI model with high feasibility. Therefore, images from people aged <10 years old were excluded from this analysis. However, additional analysis on an adapted AI model is being conducted to determine the diagnostic accuracy of the algorithm in people with hemophilia aged <10 years old.

Details on the race and ethnicity of the patients were not collected as part of this retrospective study; however, as all participating centers were located in Japan, it is likely that the majority of the images were from Asian patients. It is therefore not known whether the diagnostic precision of the AI algorithm demonstrated here would extend beyond this population. However, to date, there have been no reports of differences in joint ultrasound images based on race or ethnicity.

When comparing the algorithm diagnoses with the patients’ medical records, it should be noted that this was a retrospective observational study, and so the reliability of the bleeding records was not confirmed.

To improve the AI model, future updates could be made to increase its diagnostic accuracy while maintaining the processing speed of less than 1 second per image. The model could be updated to display the output area, which would provide suggested lesion areas to aid the physician in narrowing down the diagnostic points, and the heatmap would allow for the physicians to determine the reliability of the ROI. The heatmap, which shows the judgment of the AI model for detection of hemarthrosis and synovitis, helps the physicians to understand the AI results better and prevent misleading diagnosis in case of using the wrong areas for detection.

The establishment of ultrasound imaging conditions could improve the quality of the images and the accuracy of the AI analysis by providing uniformity. Additionally, a detailed analysis of the differences between the physician and AI model results and the identification of cause of failure for the model to detect hemarthrosis and synovitis in some cases could subsequently improve the performance of the algorithms. Finally, the model could be improved by adding in support for different imaging conditions, such as alternative procedures, equipment, and environments or using MRI and other imaging techniques.

## 5 Conclusions

These data show that utilizing AI models to assist in diagnosing hemarthrosis and synovitis could benefit clinical practice by reducing the burden on healthcare professionals, preventing oversights, and leading to earlier diagnoses and appropriate therapeutic intervention, ultimately helping people with hemophilia to achieve a healthy and active life.
